# Self-Organization and Swelling of Ruthenium-Metal Coordination Polymers with PTA (Metal = Ag, Au, Co)

**DOI:** 10.3390/polym10050528

**Published:** 2018-05-15

**Authors:** Benjamin Sierra-Martin, Manuel Serrano-Ruiz, Victoria García-Sakai, Franco Scalambra, Antonio Romerosa, Antonio Fernandez-Barbero

**Affiliations:** 1 NanoLab, Department of Chemistry and Physics, University of Almeria, 04120 Almeria, Spain; bsierra@ual.es; 2 Inorganic Chemistry Lab-CIESOL, Department of Chemistry and Physics, University of Almeria, 04120 Almeria, Spain; mserrano@ual.es (M.S.-R.); fs649@inlumine.ual.es (F.S.); romerosa@ual.es (A.R.); 3 ISIS Pulsed Neutron and Muon Source, Rutherford Appleton Laboratory, Harwell Science & Innovation Campus, Chilton, Didcot OX11 0QX, UK; victoria.garcia-sakai@stfc.ac.uk; 4 Institute of Applied Chemical Sciences, Universidad Autonoma de Chile, Santiago 8320000, Chile

**Keywords:** organometallic, ruthenium complexes, polymer dynamics, quasi-elastic neutron scattering, small-angle neutron scattering, light scattering

## Abstract

We present the internal structure and dynamics of novel coordination polymers based on two metal-containing moieties Ru-X (X: Ag, Au, Co), bridged through the phosphine PTA (3,5,7-triaza-phosphaadamantane). X-ray scattering gives the heterometallic polymer organization. Quasi-elastic neutron scattering measurements over a broad temperature range show a transition from vibrational Debye-Waller behavior to a more dynamically active state, but with rather localized motions, coinciding with the loss of structural water at around room temperature. Light scattering reveals that the polymers self-associate to form stable micro-particles in aqueous solution with a thermally driven volume transition. This is described by the Flory theory for polymers in solution, in which the polymer solvency is calculated as a function of the temperature. Polymer self-organization is further studied by small-angle neutron scattering and electron microscopy. A polymer parallel-plane model with gaps controlled by the environmental temperature is proposed.

## 1. Introduction

Organometallic polymers [1,2] historically commenced with the synthesis of poly-vinylferrocene [3]. Combination of metals and organic-polymeric frameworks leads to new polymers with superior properties, such as enhanced luminescence, extensive flame resistance, high flexibility, redox activity, and chemical sensing and electrochromic behavior [4,5,6]. Few examples of organometallic polymers are soluble in water. The first example was the metal-backbone organometallic polymer (MBOP) {[(PTA)_2_CpRuDMSO-μ-AgCl_2_-]}*_n_* (DMSO: Dimethyl sulfoxide) [7]. This polymer shows a linear structure with PTA (3,5,7-triaza-phosphaadamantane) as a metal coordinating spacer between the monometallic {CpRu(DMSO)} and {AgCl_2_} units that are bonded to the P and N_PTA_ atoms, respectively. Following on from this, a series of PTA-containing MBOPs with thermosensitive swelling was synthesized [8].

The family of heterometallic polymers [{RuCp(PTA)_2_-μ-CN-1κ*C*:2κ^2^*N*-RuCp(PTA)_2_}-μ-MX_m_]*_n_* (M: transition metal, X: halide, pseudohalide) has been extended to new systems [9]. Some of these polymers display interesting properties, such as the formation of structured microparticles and the tendency to amorphize under mild conditions. These features, in addition to their crystallinity, place these compounds between metal organic frameworks (MOFs) and infinite coordination polymers (ICPs) [10]. These systems represent promising materials for industrial and biological applications, e.g., smart catalysis, drug delivery, and chemical sensing, where a deep knowledge of the structure and dynamics is very convenient.

The aim of this paper is to study the internal structure and dynamics of coordination polymers based on two metal-containing moieties Ru-X (X: Ag, Au, Co), bridged through a tridentate phosphine (PTA). Quasi-elastic neutron scattering measurements in the powder state show the transition from vibrational Debye-Waller behavior to a more dynamically active state, but with rather localized motions, coinciding with structural water release at around room temperature. Polymers self-associate in aqueous solution, forming stable micro-particles that exhibit swelling properties, specifically a thermally controlled size transition. Self-organization is studied by photon and small-angle neutron scattering in order to sweep the meso- and nanoscales, and changes are observed as a function of temperature. Particle swelling at colloidal scale is described by the Flory theory. The nanoscopic information suggests a polymer parallel-plane model, with gaps controlled by the environmental temperature.

## 2. Materials and Methods

### 2.1. Synthesis of the Coordination Polymers

All chemicals were purchased from Sigma-Aldrich (St. Louis, MO, USA) at reagent grade and were used as received. Solvents were all degassed and distilled according to standard procedures [11]. The complexes RuClCp(PTA)_2_ and [RuCp(PTA)_2_-μ-CN-1κ*C*:2κ*N*-RuCp(PTA)_2_](CF_3_SO_3_) used as reagents were synthesized as described in the literature [12,13]. All reactions were carried out under dry nitrogen atmosphere by standard Schlenk-tube techniques.

Polymer Ru-Ag was prepared by adding 0.5 mL of a DMSO solution containing AgOTf (100 mg) to a solution of [RuClCp(PTA)_2_] (201 mg) and NH_4_Cl (20.8 mg) in 0.5 mL of DMSO. The reaction mixture was maintained at 40 °C for 1 h. The addition of *n*-hexane (3 mL) after cooling down led to Ru-Ag as a white powder, which was filtered, washed against *n*-hexane (2 × 1.5 mL), and dried under vacuum [14]. Yield: 96%. The Ru-Au polymer was obtained as follows: a solution containing 29.9 mg of K[Au(CN)_4_] and 5 mg of KCN in 1 mL of H_2_O was added to a solution of [RuClCp(PTA)_2_] (80 mg) in 5 mL of H_2_O. The resulting solution was kept at 90 °C for 5 min before being cooled to room temperature and filtered. The solvent was removed under vacuum to give a yellow powder, which was dissolved in DMSO (1 mL). Yellow crystals suitable for X-ray diffraction were obtained after 2 weeks. Yield: 57%. Polymer Ru-Co was prepared by addition of a mixture of anhydrous CoCl_2_ (114.3 mg) and NH_4_Cl (47.1 mg) in 10 mL of methanol to a solution of [RuCp(PTA)_2_-μ-CN-1κ*C*:2κ*N*-RuCp(PTA)_2_](CF_3_SO_3_) (1.0 g) in 50 mL of acetone. The obtained blue powder was filtered and dissolved at 90 °C in 10.7 mL of a DMSO/H_2_O 10/1 mixture. The final solution was cooled down and kept at 25 °C for 2 days. The light-green crystals were filtered and dried under air [13]. Yield: 40%.

### 2.2. X-ray Structure Determination

Data were collected on a Bruker (Billerica, MA, USA) APEX CCD area detector diffractometer (XDIFRACT service of the University of Almeria, Almeria, Spain) using MoKα radiation (λ = 0.71073 Å). Empirical absorption corrections were applied. The structure was solved by direct methods (SIR97) and refined by full matrix least squares on *F*^2^ (SHELX 97). Anisotropic thermal factors were assigned to all the non-hydrogen atoms. For the Ru-Ag polymer, the cyclopentadienyl C atoms were statistically distributed around the Ru atom. All the H atoms, other than those bonded to O30 and cyclopentadienyl C atoms, were located at the calculated positions and were not refined. In the case of Ru-Au, all the H atoms except those bonded to the water molecules were located at the calculated positions and not refined. Moreover, the water molecule H atoms were located by Fourier difference synthesis. All diagrams were generated by the SHELXL program. The crystallographic parameters of the three polymers are summarized in Tables S1–S3.

### 2.3. Photon Correlation Spectroscopy (PCS)

This technique is used to quantify polymer self-assembly at colloidal length scales. A linearly polarized incident light beam reaches the sample and induces dipolar moments μ, related to a first approximation to the incident electrical field through the polarization tensor, μ(*t*) = α*E*(*t*). The dipolar moments give out electromagnetic radiation at a particular scattering angle, θ. The fluctuation of the scattered intensity is a consequence of the system dynamics and is usually analyzed through the intensity autocorrelation function *g*_int_(*q*, τ) = <*I*(*t*)*I*(*t* + τ)> = lim 1/*T* ∫*I*(*t*)I(*t* + τ) *dt*, at a given wave vector *q* = (4π*n*/λ)sin(θ/2), with λ being the wavelength of light and *n* the medium refractive index [15]. The field autocorrelation function *g*_field_(*q*, τ) = <*E*(*t*)*E*(*t* + τ)> is calculated from *g*_int_ by means of the Siegert relationship, *g*_field_(*q*, τ) = 1 + γ|*g*_int_(*q*, τ)|^2^. γ depends on the particular optic arrangements, optimized for detecting the intensity of fluctuations. The number of coherence areas on the detector sets to ∼1–2, further implying γ ≈ 1 [15]. The autocorrelation function decays as *g*_field_ (*q*, τ) = exp(−Γτ)(1 + ∑*a*_n_τ^n^/*n*!). This series expansion allows extraction of dynamic information. The decay time Γ = −*d*/*d*τ ln[*g*_field_(τ)]|_τ→0_ = <*D*>*q*^2^, contains the mean diffusion coefficient of the scattering units, <*D*>. The size polydispersity is usually estimated as (*a*_n_/Γ^2^) [16].

A 5 mW He-Ne vertical-polarized laser (λ = 632.8 nm) illuminates the sample in a cylindrical cell located at the center of an index matching chamber to block undesired reflections. A photomultiplier harvests the photons scattered at 40° and feeds a Malvern electronic photo-correlator (Malvern, UK). The temperature is controlled to within ±0.1 °C by a Peltier cell device (Malvern, UK) and water flowing through an external bath chamber. Colloidal structure contributions and multiple scattering effects are minimized using diluted dispersions. Results are the average of five measurements. The mean diffusion coefficient is converted into mean particle size using the Stokes-Einstein equation for spherical particles, once corrected for temperature and solvent viscosity.

### 2.4. Neutron Scattering

The structure and dynamics of the polymers are investigated by neutron scattering. Neutrons bombarded onto a sample are scattered by the atomic nuclei, with the possibility of exchanging momentum and energy. The momentum transfer, *Q* = (4π/λ)sin(θ/2), is related to the spatial scale probed, and the energy transfer, ℏω, provides information about the time scale of the dynamics. All the information about the system is contained within the so-called scattering function *S*(*Q*,ω). Neutron scattering measures the double differential scattering cross-section *d*^2^σ/*d*Ω*dE*, which depends on the strength of the interaction between a particular isotope and the neutron, known as the scattering cross-section σ. The latter has two contributions, coherent and incoherent, yielding the measurements of the *S*_coh_(*Q*,ω) and *S*_inc_(*Q*,ω) scattering functions, which are used primarily to obtain information about structure and dynamics, respectively. In this work, we use two neutron scattering techniques, Small-angle neutron scattering (SANS) and Quasi-elastic neutron spectroscopy (QENS), to probe the aforementioned functions.

#### 2.4.1. Small-Angle Neutron Scattering

Measurements are carried out using the LOQ spectrometer at the ISIS Pulsed Neutron and Muon Source, Rutherford Appleton Laboratory (Didcot, UK) [17]. Structural information comes from the coherent term of the scattering function, and thus it is of paramount importance to minimize the incoherent contribution; for this, we use D_2_O as the polymer solvent instead of light water. Neutrons from a 25 K liquid hydrogen moderator are collimated prior to striking the sample position at 11.1 m from the moderator. The beam size at the sample is 8 mm in diameter. The secondary flight path after the sample is an evacuated tank with the detector (64 cm × 64 cm 3He-CF4 filled ORDELA “area” with 5-mm resolution) located 15.15 m from the moderator. The incident beam wavelength is selected at 6 Å with a spread of 10%, yielding an accessible scattering *Q*-range of 0.01–0.34 Å^−1^. The detector efficiency is corrected using H_2_O as a pure incoherent scatterer. Data are corrected for the direct beam (using a Cd sheet at the sample position), for the empty cell, and for D_2_O scattering, as well as for sample transmission. Any incoherent scattering contribution due to hydrogen is corrected by assuming the additivity of the incoherent scattering between the polymer and D_2_O. The polymer concentration in D_2_O was 4 wt %. All samples are contained in 1-mm-path Hellma quartz cells and equilibrated for 15 min under a controlled temperature, prior to data recording. The data were treated using ISIS provided software (Didcot, UK).

#### 2.4.2. Quasi-Elastic Neutron Spectroscopy

QENS measurements are carried out using the IRIS time-of-flight backscattering spectrometer in the ISIS Pulsed Neutron and Muon Source, Rutherford Appleton Laboratory (Didcot, UK) [18]. Neutrons from a 25 K liquid hydrogen moderator are transported down 36 m of neutron guide towards the sample position. A band of neutron wavelengths around 6.66 Å are selected using a neutron chopper that impinge on the sample. Scattered neutrons are then energy-analyzed by means of Bragg reflection from pyrolytic graphite single-crystal arrays in close to backscattering geometry. The analyzed neutrons are then detected by a bank of 51 ZnS scintillators that cover scattering angles between 25° and 160°. IRIS can access a *Q*-range of 0.4 to 1.85 Å^−1^ and has an energy resolution of 17.5 μeV. The technique called QENS refers to the possibility of measuring at high energy resolutions, probing signals close to the elastic line (no energy exchange), giving access to low-energy modes like translational and rotational modes, and slower segmental relaxations in polymers. Measurements are typically made with largely hydrogenated samples, since the incoherent neutron scattering cross-section is much higher than that of other common elements such as carbon, oxygen and nitrogen. For the polymers in this paper, the ratio σ_inc_(H)/σ_inc_ (total polymer) is estimated to be 0.997, and the scattering is then mainly dominated by the incoherent radiation from H atoms. In this way, we can infer the dynamics of the polymeric chains to which the H atoms are attached. The measured incoherent scattering function is directly related to the self-dynamic correlation function.

Samples are placed in standard annular aluminum containers with an annular gap chosen to minimize multiple scattering (typical measurements are for a 10% scatterer). A vanadium standard, a pure incoherent scatterer, is used to correct for detector efficiency. The resolution of the instrument is measured using the sample at 4 K. The sample temperature is controlled using a top-loading closed-cycle refrigerator.

## 3. Results and Discussion

### 3.1. Structure of the Coordination Polymers

**Scheme 1:**
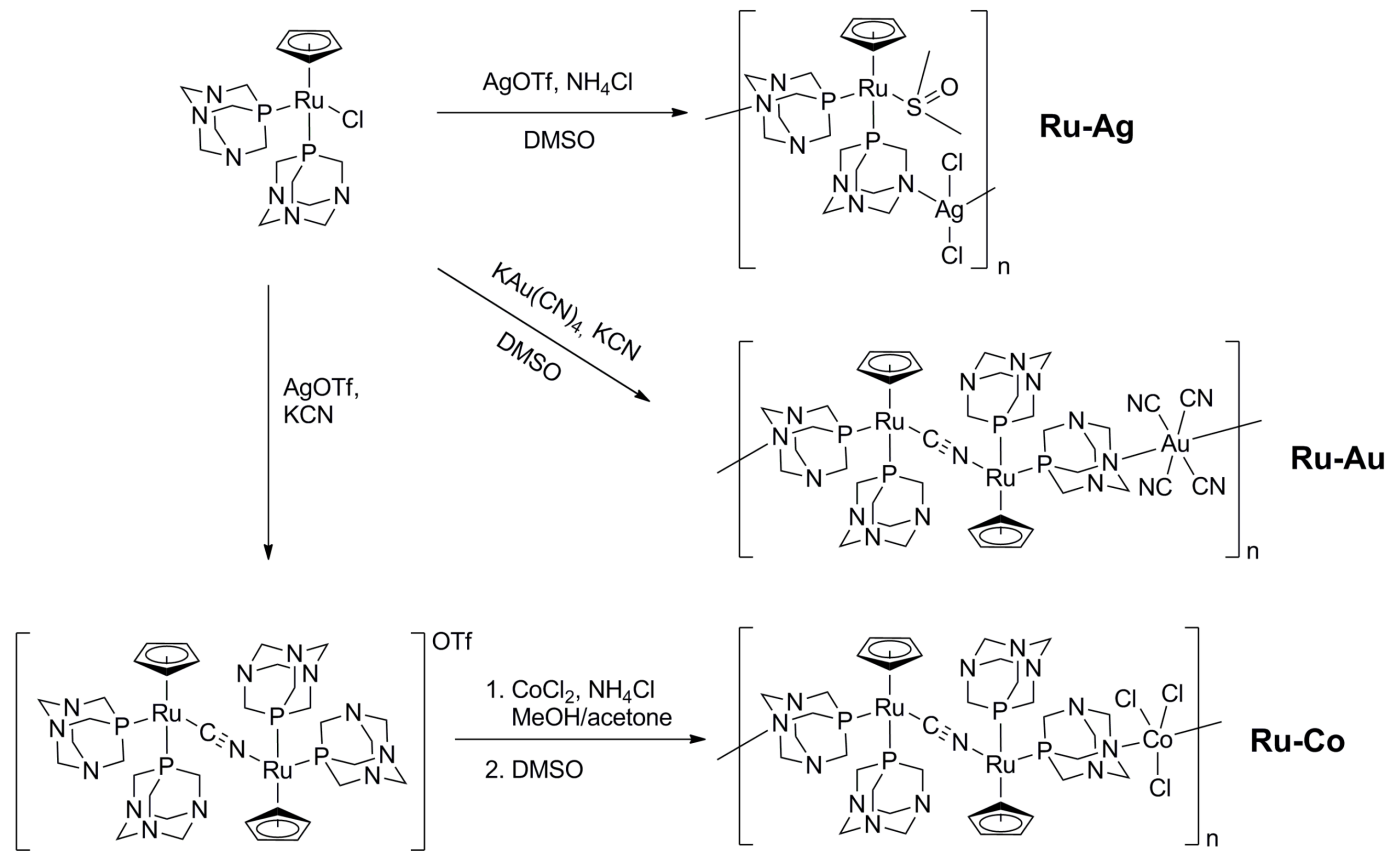
Synthesis of the coordination polymers.

The organometallic polymers {[(PTA)_2_CpRuDMSO-μ-AgCl_2_-]}*_n_* (Ru-Ag) and [{RuCp(PTA)_2_-μ-CN-1κ*C:*2κ^2^*N*-RuCp(PTA)_2_}-μ-Au(CN)_4_]*_n_* (Ru-Au) are obtained by reacting [CpRuCl(PTA)_2_] with AgCl or [Au(CN)_4_]^−^, in each case, in DMSO containing water (Scheme 1). The polymer *cis*-{[{RuCp(PTA)_2_-μ-CN-1κ*C*:2κ^2^*N*-RuCp(PTA)_2_}-μ-CoCl_3_]}*_n_*·{[RuCp(PTA)_2_-μ-CN-1κ*C*:2κ^2^*N*-RuCp(PTA)_2_]Cl}_0.5*n*_·(15H_2_O)*_n_* (Ru-Co) is prepared from reaction of the dimeric ruthenium complex [RuCp(PTA)_2_-μ-CN-1κ*C:*2κ^2^*N*-RuCp(PTA)_2_](CF_3_SO_3_) with CoCl_2_ in DMSO:H_2_O (10:1 volume). The polymer crystallization of them provides single crystals for X-ray determination.

**Figure 1:**
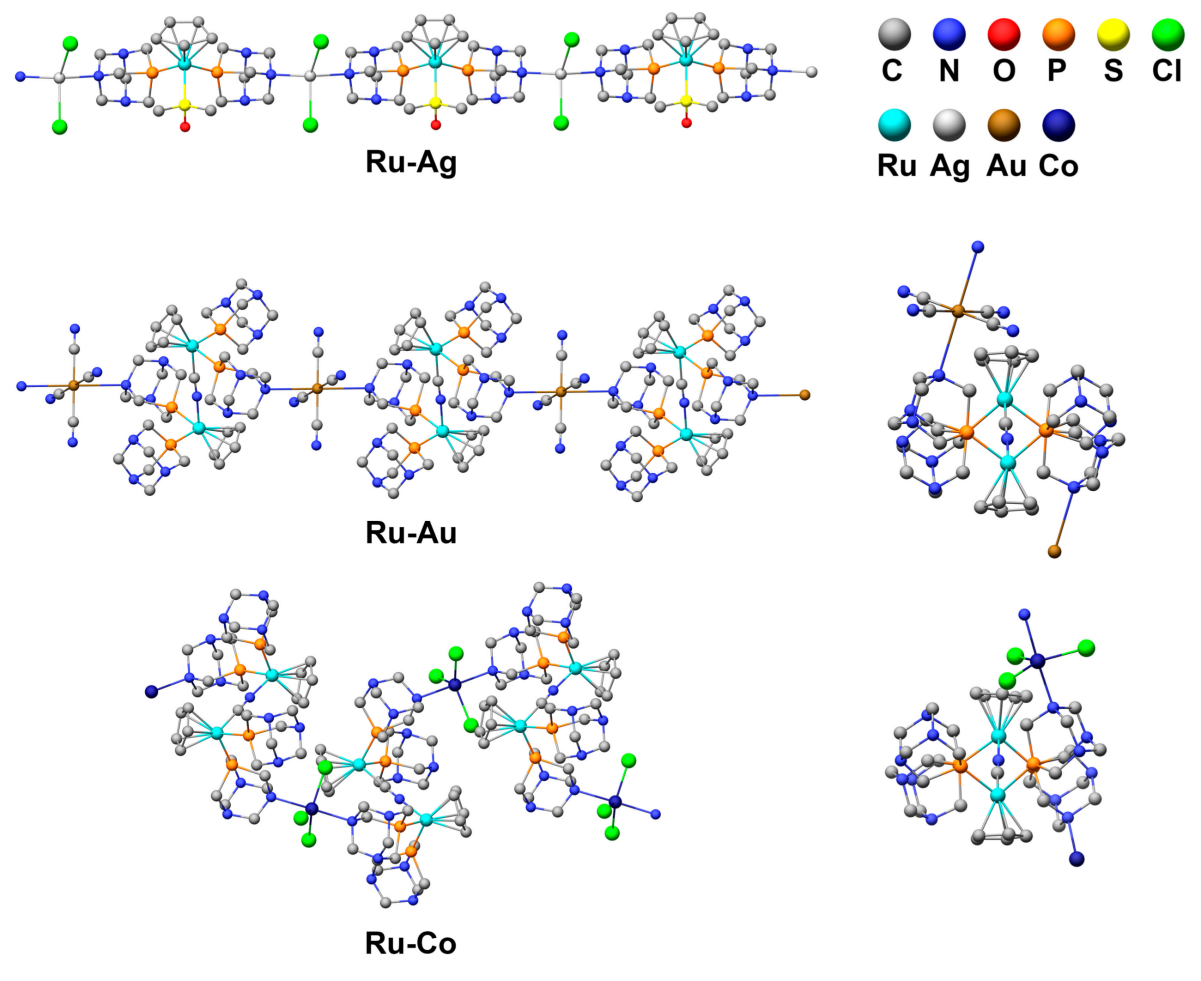
X-ray molecular structure of the coordination polymers.

The Ru-Au polymer is formed by linear chains of alternating {CpRu(μ-CN)RuCp}^+^ moieties and [Au(CN)_4_]^−^ anions bridged by PTA ligands acting as tethering units (Figure 1). The moiety {CpRu(μ-CN)RuCp}^+^ contains two ruthenium atoms linked by a CN ligand. Both Cp and PTA ligands are disposed in opposite direction as imposed by the “i” symmetry element at the middle of the C–N bond length. For polymer Ru-Co (Figure 1 bottom), its cell asymmetric unit contains the heterometallic moiety {(RuCp(PTA)_2_-μ-CN-1κ*C*:2κ^2^*N*-RuCp(PTA)_2_)-μ-CoCl_3_}, as the basic repeating unit, plus one {RuCp(PTA)_2_-μ-CN-1κ*C*:2κ^2^*N*-RuCp(PTA)_2_}^+^ unit, and 15 water molecules (Figure 1). The Ru-Au polymer is constituted by the {Cp(PTA)_2_Ru-μ-CN-RuCp(PTA)_2_}^+^ moiety, similarly to the Ru-Co, but linked through the N atoms of the PTA to {Au(CN)_4_}^−^ groups instead to {CoCl_3_}^−^. On the other side, the Ru-Ag polymer is structurally different to Ru-Au and Ru-Co. It displays a molecular structure consisting of linear chains of alternating [CpRu(DMSO-κ*S*)]^+^ cations and [AgCl_2_]^−^ anions, bridged by PTA ligands (Figure 1).

### 3.2. Polymer Visualization and Self-Assembly

**Figure 2:**
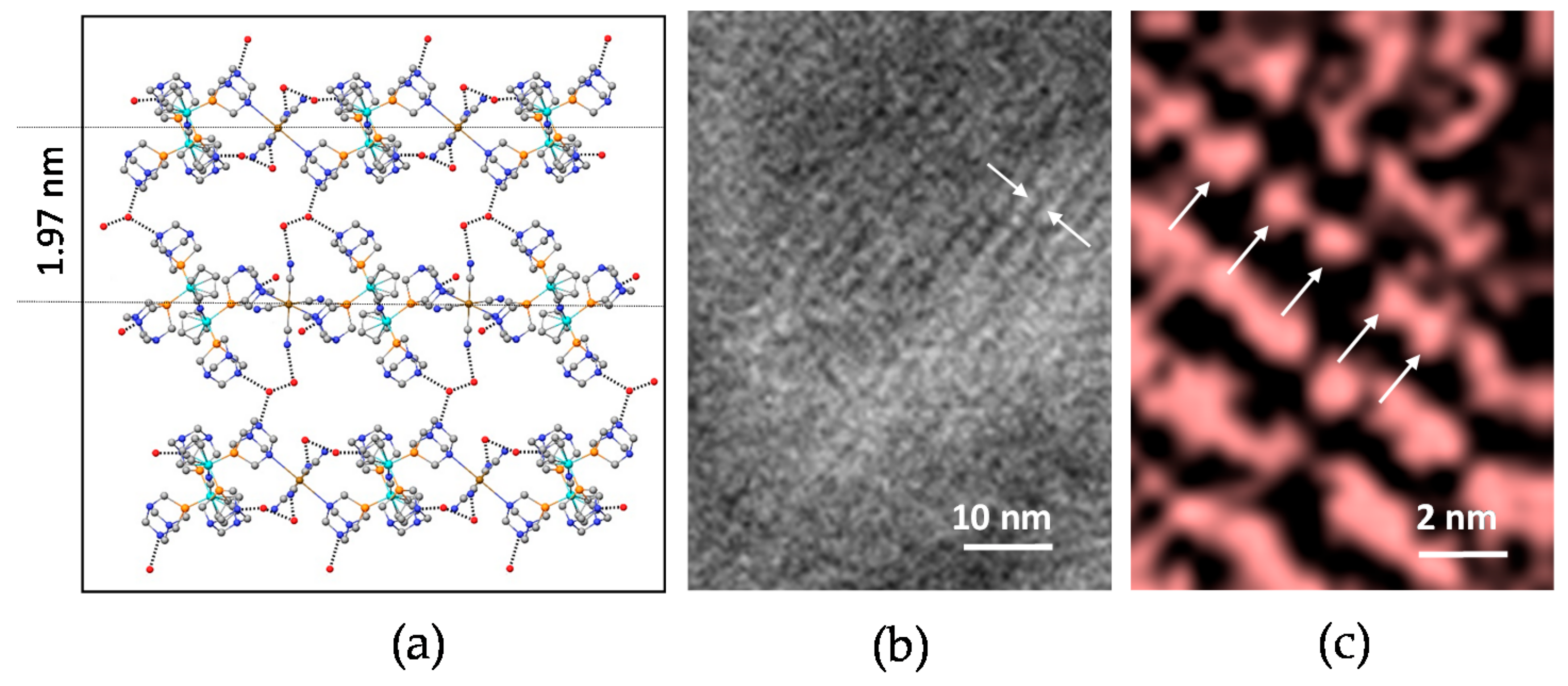
(**a**) Spatial distribution of the Ru-Au polymer showing the hydrogen bonding network connecting the polymeric chains via PTA nitrogen. Red beads represent structural water molecules bridged through hydrogen bonds; (**b**) TEM picture showing parallel stripes that correspond to polymer rows. The gap between two adjacent polymer rows are indicated by arrows; (**c**) Detailed zoom TEM picture showing single units in a row corresponding to the monomers. Arrows point to different monomer units spread on a single polymer row.

X-ray data gives insights into the molecular organization of the polymer chains, as shown for the Ru-Au system in Figure 2a [8]. Polymeric units spread along chains and are held together in a 3D structure by electrostatic and hydrogen bond interactions. Polymer chains interact with adjacent chains, self-organizing into parallel plane stacks separated by a distance of 1.972 nm. Hydrogen bonds connect the polymeric chains via PTA nitrogen with structural water molecules bridged through the hydrogen bonds. Transmission Electron Microscopy (TEM) (JEOL, Akishima, Japan) allows the visualization of the mesoscopic structure. Water-dissolved Ru-Au polymer was deposited on Fomvart and dried at 40 °C for 12 h before sample observation. Regular stripes in Figure 2b with gaps of about 2 nm correspond to the polymer rows, consistently with the X-ray information. Figure 2c zooms local details from the picture and shows single units in a row, which corresponds to the monomer units, separated by a distance of about 2 nm.

### 3.3. Polymer Dynamics

**Figure 3:**
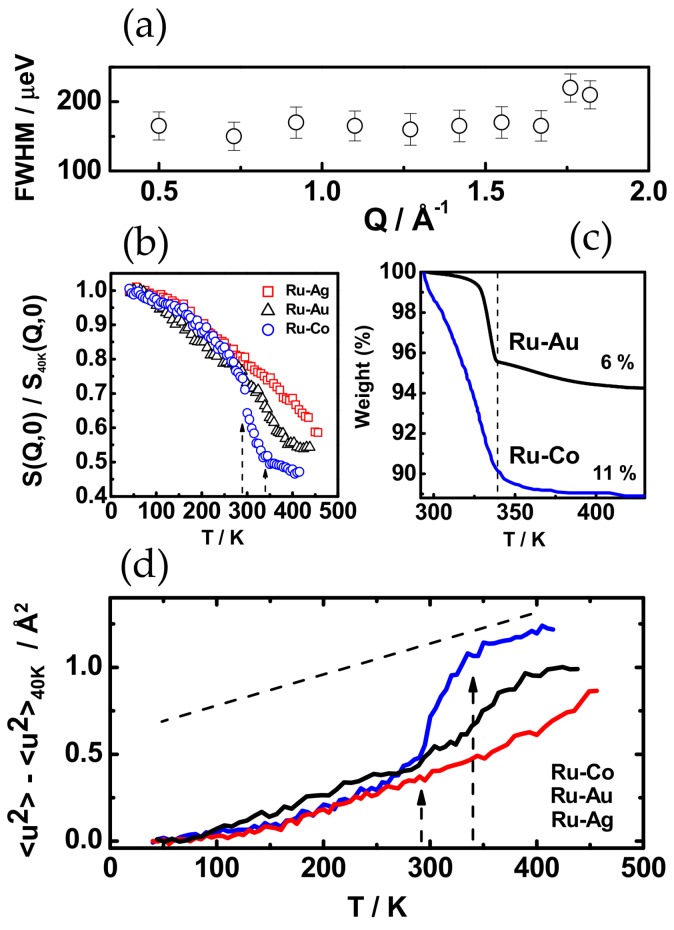
(**a**) *Q*-independence plot confirms little structural contribution to the signal for Ru-Au; (**b**) Normalized elastic scattering intensity for Ru-Ag, Ru-Au and Ru-Co; (**c**) Thermo-gravimetric curves taken at 2 °C/min heating rate. Mass loss corresponds to about 6% for Ru-Au and 11% for Ru-Co; (**d**) Mean square displacement <*u*^2^> calculated for the tree polymers.

As mentioned previously, the samples used are fully hydrogenated, and thus we expect very little structural contribution to the observed signal. A pseudo-check can be done by integrating the elastic intensity (within the resolution of the IRIS spectrometer, 17.5 µeV) and plotting it as a function of *Q*. Figure 3a checks this aspect for one of the three polymers.

The incoherent elastic neutron-scattering structure function *S*_inc_(*Q*,0) is a convenient way to monitor the onset of molecular motions. It is recorded by doing a so-called ‘fixed window scan’ [19], where data is taken at given intervals of increasing temperature. When the scattered intensity is normalized to that at very low temperature, in this case 40 K, where no motions are expected within the timescale that IRIS can probe, and plotted as a function of temperature, distinct changes in slope can indicate important features. Figure 3b shows the data for the three polymers at an average *Q* value of 1.36 Å^−1^. An initial decreasing drift is observed at low temperature [20], becoming sharper around 280 K, the latter being particularly pronounced for Ru-Co. At about 340 K, the decreasing rate slows down, and the structure function continues to evolve, similarly to at the beginning. This behavior is almost imperceptible for the Ru-Ag polymer.

The vibrational spatial scale of the scatters (in this case mostly hydrogen) is analyzed in terms of the mean square displacement <*u*^2^>, evaluated from the equation ln[*S*_inc_(*Q*,0)/*S*_inc_(*Q*,0)_40 K_] = −⅓[<*u*^2^(*T*)> − <*u*^2^(40 K)>] *Q*^2^. Figure 3d shows <*u*^2^ (*T*)> − <*u*^2^(40 K)> plotted against temperature for the three coordination polymers. An expected Debye-Waller linear dependence is observed at low temperatures, where <*u*^2^> increases linearly with respect to the temperature for harmonic oscillators. Deviations from linearity indicate the onset of anharmonic vibrations and/or relaxation processes in the polymer [21]. At about 280 K, <*u*^2^> increases drastically for Ru-Co and Ru-Au, and then indicates the onset of a relaxation.

**Figure 4:**
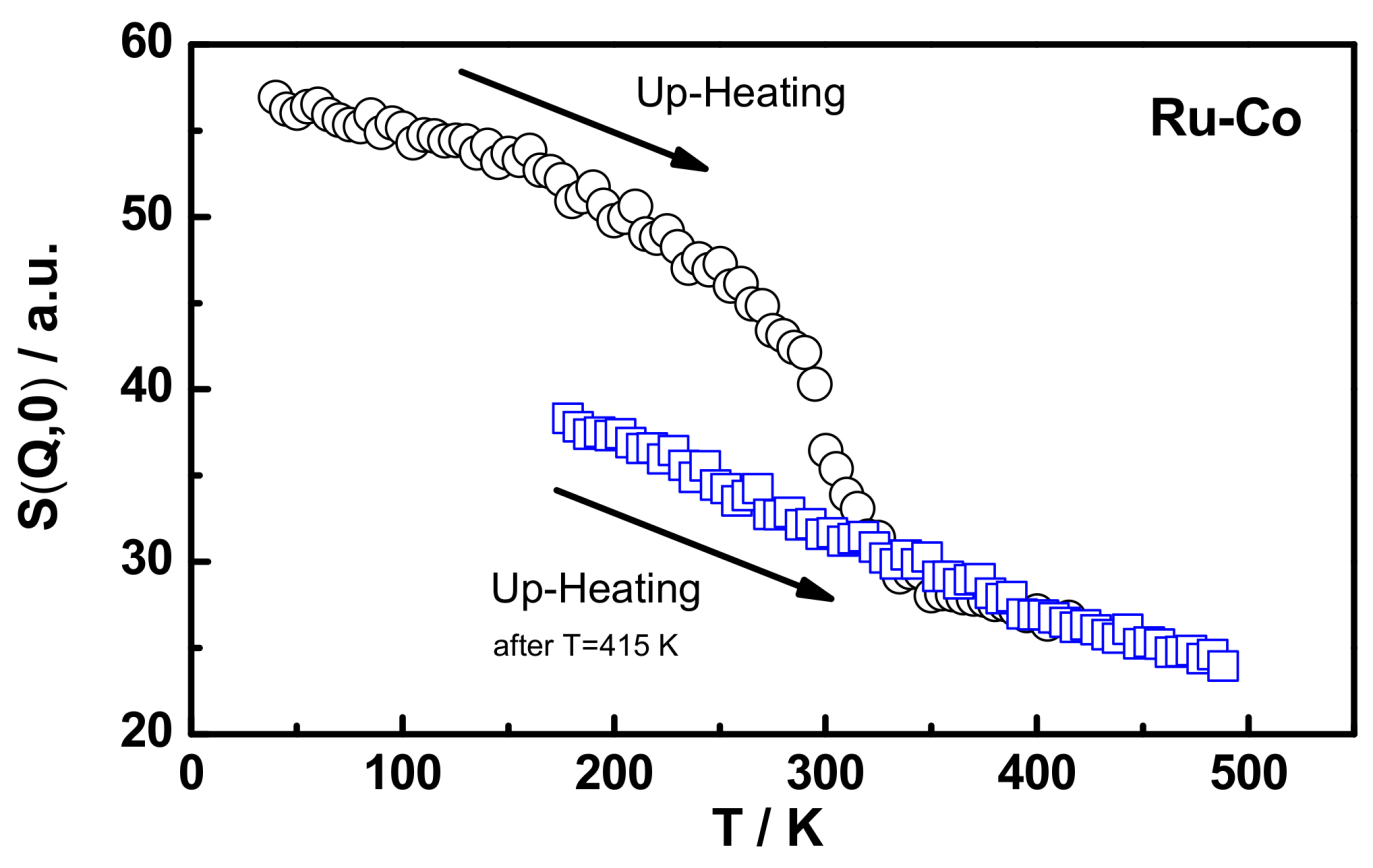
Hysteresis of the Ru-Co polymer due to the loss of structural water, observed by QENS.

In order to gain more insight into the relaxations, thermo-gravimetric experiments were carried out for the systems Ru-Au and Ru-Co. Figure 3c shows a loss of material for both systems at 280 K, the temperature that triggers the polymer relaxation. The weight loss rate moderates at ~340 K, just at the point where, especially for the Ru-Co, the mean-square displacement changes its trend again. Lost weight corresponds to 11% and 6% for Ru-Co and Ru-Au, respectively, coinciding with the amount of structural water calculated from X-ray. The structural water links the polymer chains through hydrogen bounds, keeping rigid the polymer structure. As temperature increases, these bonds break, and the structural water is released. This fact provokes the observed increase of polymer dynamics. The release of water molecules generates vacancies within the polymer structure that could allow their substitution by molecules with similar size. To check that the final structure is water-free, we cooled the Ru-Co sample down back to 170 K, equilibrated and reheated, as shown in Figure 4. The temperature required for eliminating the water from the solid structure of the complexes is determined by the hydrogen bond strength among water molecules and the polymer. This, in turn, is related to the hydrogen bond distance. For Ru-Ag, the shortest distance between two H atoms at adjacent polymer chains mediated by a water molecule is 2.699 Å [7]. This distance is longer than that corresponding to strong hydrogen bonds, but shorter than the sum of the van der Waals radii [22]. The above-mentioned distance is 2.723 Å for Ru-Au and 2.817 Å for Ru-Co [5]. Taking into account these distances, the temperature required to release the structural water should increase following the series Co-Au-Ag, as experimentally observed in Figure 3.

### 3.4. Assembly of Polymers in Water: Colloidal Scale

**Figure 5:**
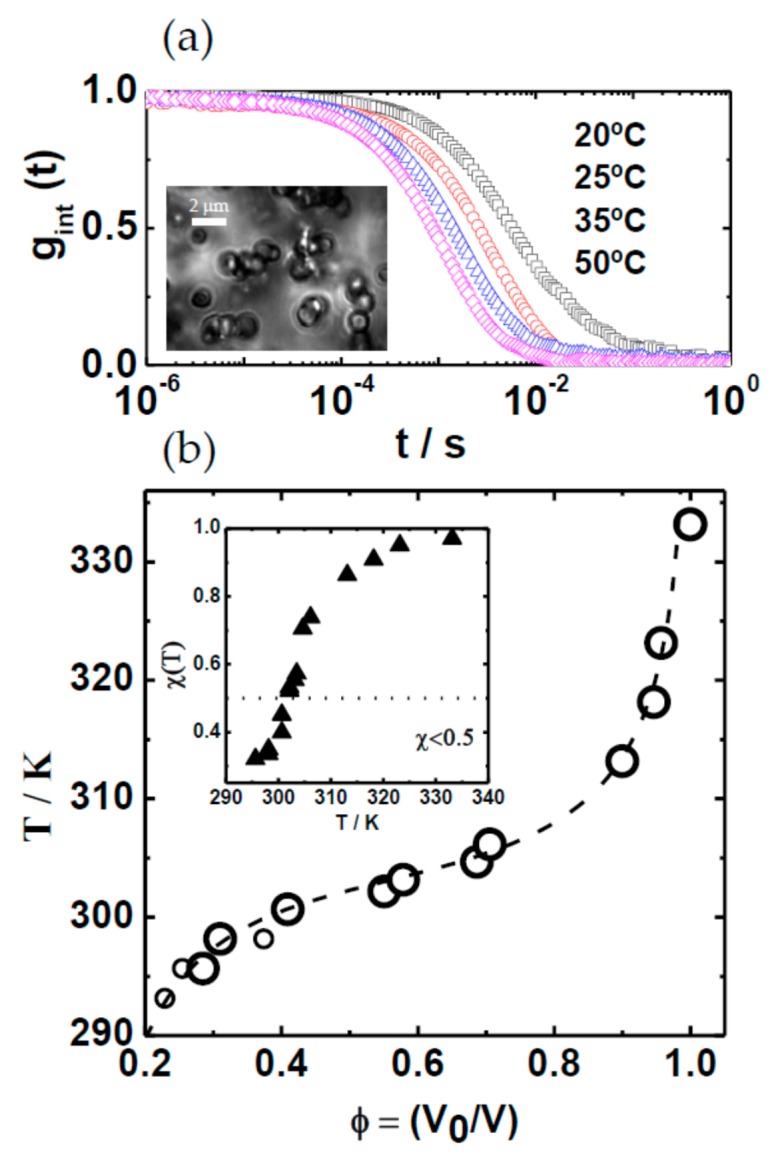
(**a**) Intensity autocorrelation function for the colloidal particles at different temperatures. Inset: Optical microscopy of Ru-Au polymer microparticles; (**b**) Phase diagram for the Ru-Au colloidal particles. The dashed line is a guide to the eye. Inset: Flory’s solvency function, χ(*T*).

The Ru-Ag and Ru-Au polymers dispersed in water retain their structure and associate to form stable micro-particles (Figure 5a, inset). PCS is used to observe the polymer self-assembling at colloidal scale. The intensity autocorrelation function for Ru-Au is plotted in Figure 5a at different temperatures. Mean particle diffusion coefficients calculated from the relaxation slopes are converted into mean particle size. Particles within the range 800–1200 nm swell and shrink in response to the environmental temperature. Figure 5b plots the *T*-*V* diagram (temperature vs particle volume fraction), with a transition between swollen and collapsed states found at ~305 K.

This behavior resembles the organic poly-*N*-isopropylacrylamide (poly-NIPAM) microgel, a classical system exhibiting temperature-controlled particle swelling [23]. Flory’s theory is considered to model particle swelling, as previously used for poly-NIPAM. At equilibrium, the solvent chemical potential is equal inside and outside the particle gel, and no net transfer of solvent takes place across the interface. A mixing osmotic term, π_m_, accounts for the polymer solubility. It shows an ambivalent behavior, balanced by the value of the Flory solvency parameter. For high polymer solubility, π_m_ tries to swell the gel, while for poor solvents, π_m_ contributes to the collapse. Once the temperature sets the polymer solubility, the particle volume evolves to reach the thermodynamic equilibrium according to equation π_m_ = (−*N*_A_*kT*/*υ*_s_) [ϕ + ln(1−ϕ) + *χ*(*T*)ϕ^2^] = 0, where *N*_A_, *k* and *υ*_s_ are the Avogadro number, Boltzmann constant and solvent molar-volume, respectively. *T* is the absolute temperature and ϕ = *V*_0_/*V* is the particle volume fraction. Flory solvency is expressed as *χ*(*T*) = *Z* [ε_ps_ − (ε_pp_ + ε_ss_)/2]/*kT*, with *Z* being the coordination number of a polymer segment and ε_ij_ the interaction energies between polymer (p) and solvent (s) molecules. The first two terms in bracket at the equilibrium equation account for the increase of entropy when solvent and polymer mix. The third term, including the polymer solvency, is of enthalpic nature, and describes the polymer-solvent interaction.

*χ*(*T*) is calculated from Flory’s model and is plotted in the inset of Figure 5b. Values evolve from about 0.3 to 1 from swollen to shrunk particles, consistent with the switching between good quality solvent (*χ* < 0.5) and poor solvent (*χ* > 0.5). We argue that the temperature dependence arises from the hydrophilic and hydrophobic character of the PTA and cyclopentadiene units, respectively. Below the transition temperature, particles are swollen because of the high solubility of PTA [9]. Nitrogen atoms in PTA are bridged through extensive hydrogen bonding to the water molecules. In addition, water molecules form clusters around the hydrophobic Cp ligands [9]. Above the transition temperature, the water-amine hydrogen bonds are disrupted and the attractive hydrophobic interactions between the polymer chains become dominant; as a result, the polymer chains collapse, expelling water from the particles [24]. To the best of our knowledge, the temperature-dependent assembly is not usual in this kind of polymer. In our systems, the behavior relies on the interaction of the hydrophobic and hydrophilic groups with the solvent. Thus, the swelling process is highly dependent on the polymer’s chemical nature and may be not directly extended to other systems.

### 3.5. Assembly of Polymers in Water: Nano Scale

**Figure 6:**
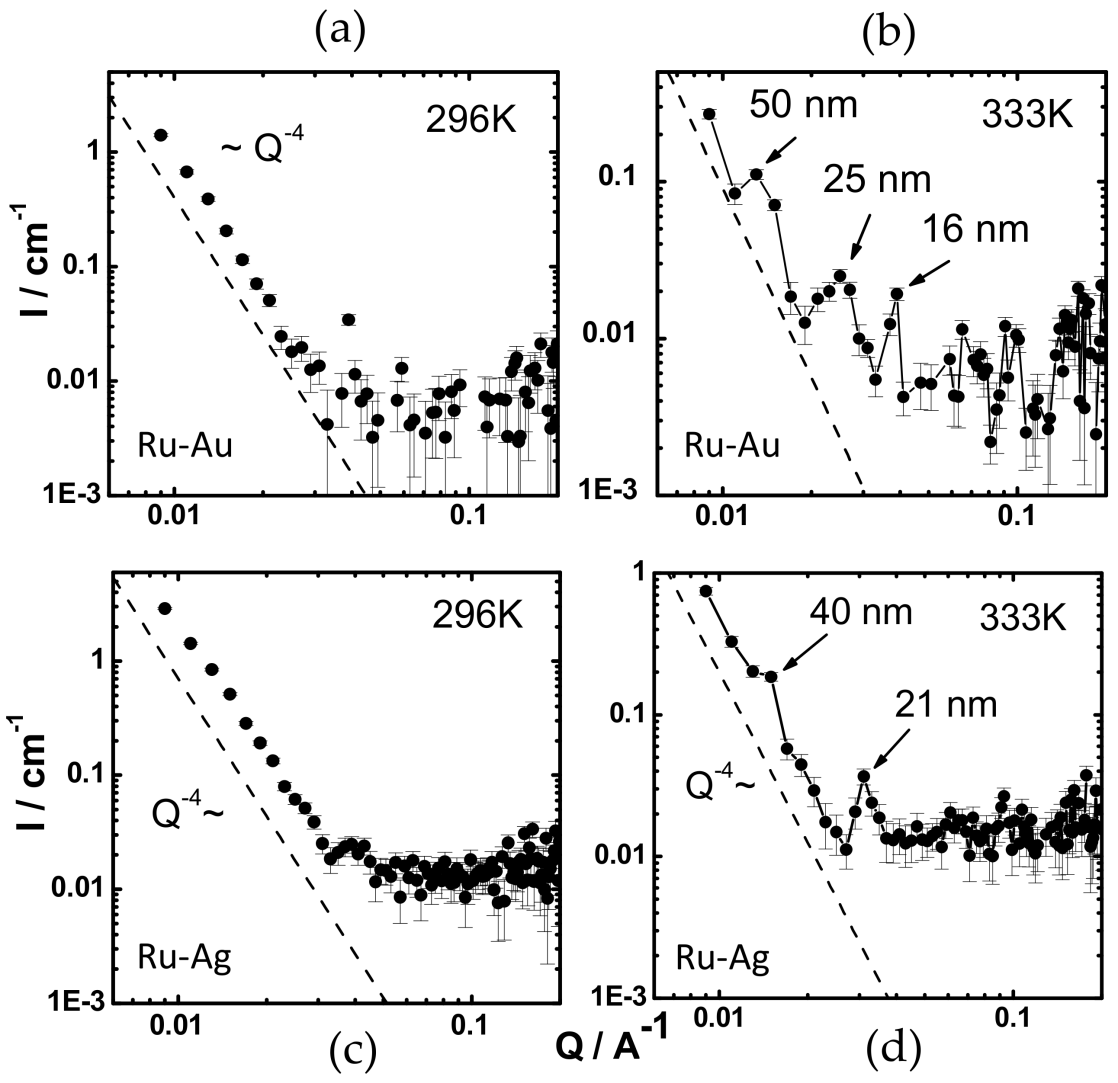
SANS intensities for Ru-Au (**a**,**b**) and Ru-Ag (**c**,**d**) polymers in D_2_O. Profiles correspond to swollen (296 K; (**a**,**c**)) and collapsed (333 K; (**b**,**d**)) states.

Small-angle neutron scattering is used to investigate the internal structure of the polymer network along the swelling [25,26]. The neutron scattered intensity *I*(*Q*) is obtained at a wave-vector transfer *Q* = (4π/λ)sin(θ/2). *I*(*Q*) in absolute scale is given by *I*(*Q*) = *I*_obs_(*Q*) + *I*_inc_(*Q*), where *I*_obs_(*Q*) and *I*_inc_(*Q*) are the observed and the incoherent scattered intensities, respectively. SANS intensities are plotted in Figure 6 for Ru-Au and Ru-Ag polymers dispersed in D_2_O. Spectra correspond to swollen (296.15 K) and collapsed (333.15 K) particles. The measured scattering intensity decreases monotonously with *Q* for swollen particles, well described by a −4 exponent power law. For colloids, the particle form factor P(*Q*) contributes significantly to the scattering intensity. The Porod contribution to P(*Q*), arising from the particle surface, dominates the scattering profile leading to *I*(*Q*) ~ *Q*^−4^, as experimentally found.

**Figure 7:**
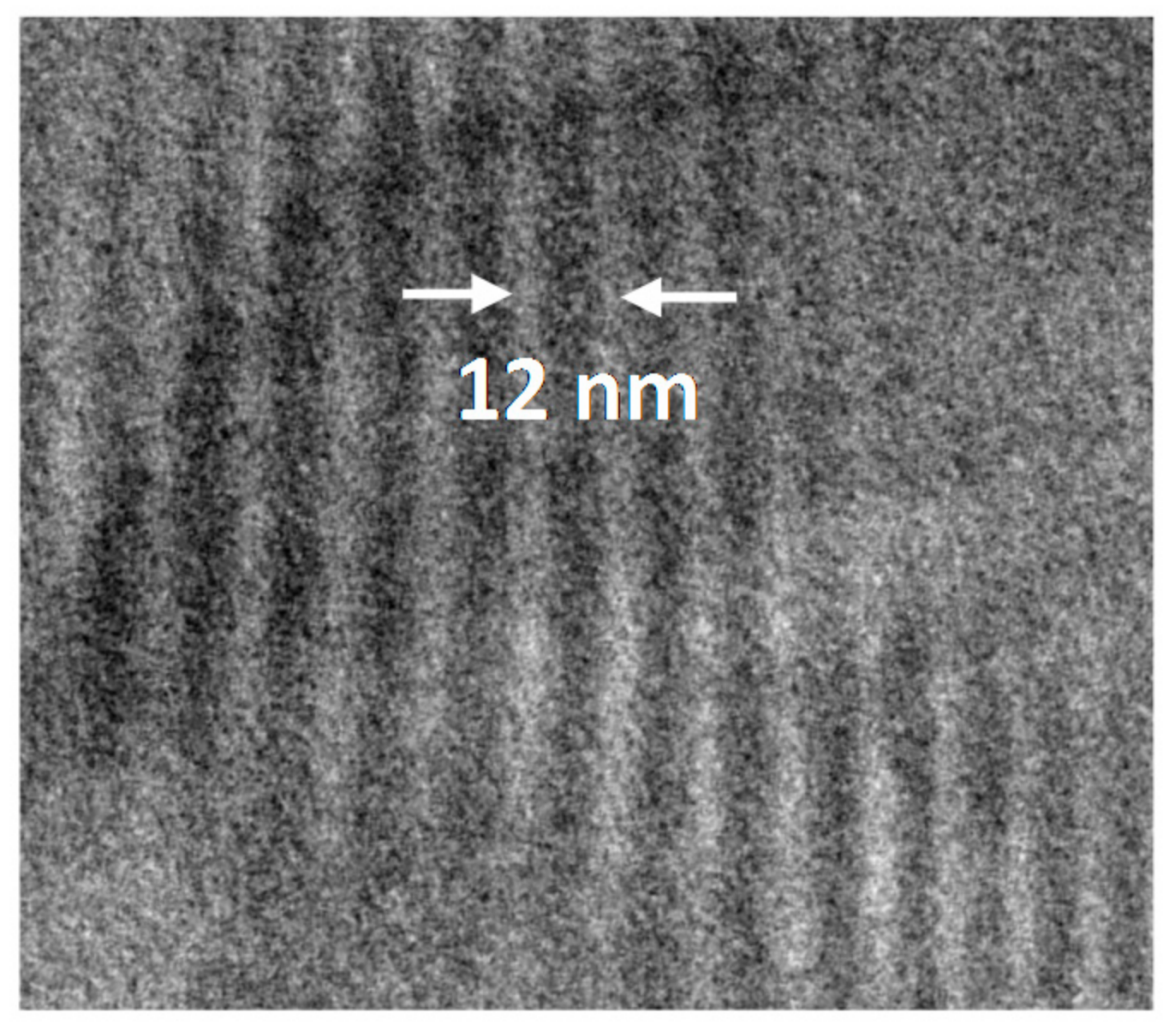
Inter-stripes gap as estimated by TEM. It is consistent with the characteristic lengths determined by SANS for collapsed state.

Upon raising the temperature, the particles collapse, they release water, and the polymer density increases and self-reorganizes. Figure 6 shows the peaks associated to the characteristic lengths calculated from ξ = 2π/*Q*_peak_. For the Ru-Au polymer, the peaks lead to lengths of around 16, 25 and 50 nm, approximately multiples of 2. This finding is consistent with a constant-gap parallel-planes model. The same behavior is observed for Ru-Ag in D_2_O, with peaks at characteristic lengths of 21 and 40 nm. The TEM picture in Figure 7 shows these parallel planes, separated by a constant gap of 12 nm for Ru-Au, which fits the lengths found by SANS. In conclusion, for swollen particles, no internal structure exists, or else it is simply not detected, within the *Q* observation window; while for collapsed particles, a parallel-plane structure is revealed with neutron scattering and TEM. As a final remark, it is interesting to point out that the Ru-Ag and Ru-Au systems show thermal swelling, but they do not both manifest the same ability to release the water in the polymer structure. This indicates that the structural water studied by QENS is not a determining factor influencing the ability of these systems to swell or de-swell.

## 4. Conclusions

We study the internal structure and dynamics of new coordination polymers based on two metal-containing moieties Ru-X (X: Ag, Au, Co), bridged through the water-soluble phosphine PTA (3,5,7-triaza-phosphaadamantane). Quasi-elastic neutron scattering displays changes for the hydrogen dynamics as the temperature increases, from vibrational Debye-Waller behavior to a more dynamically active state. The change of the proton mean square displacement is pronounced for the Ru-Co polymer, whereas it is almost imperceptible for Ru-Ag. This change, found at room temperature for polymers in the powder state, is due to the loss of structural water contained in the polymer, consistent with themogravimetric analysis. The coordination polymers self-associate in aqueous media to form stable micro-particles that interestingly exhibit a temperature-dependent size transition. The swelling is well described by the Flory theory. The polymer solvency is then calculated as a function of the temperature. Internal structure along the thermal-driven size transition is studied by small-angle neutron scattering. Peaks on the SANS spectrum for the collapsed state disappear when the polymers swell. This finding is consistent with a parallel-plane model in the internal structure. As a final remark, we point out that the vacancies originated by the release of structural water could be filled with molecules of interest for catalysis. In addition, the swelling properties can be exploited for temperature-tuning catalytic reactions.

## Supplementary Materials

The following are available online at http://www.mdpi.com/2073-4360/10/5/528/s1; Table S1: Crystallographic parameters for the coordination polymers Ru-Au, Ru-Ag and Ru-Co, Table S2: Selected bond lengths for Ru-Ag, Ru-Au and Ru-Co, Table S3: Selected bond angles for Ru-Ag, Ru-Au and Ru-Co.
