# Supplementary material for: Self-Organization and Swelling of Ruthenium-Metal Coordination Polymers with PTA (Metal = Ag, Au, Co)
Source: Polymers (Basel). 2018 May 15;10(5):528. doi: 10.3390/polym10050528 (PMC6415399; doi:10.3390/polym10050528)
Supplement: Supplementary file 1 [file polymers-10-00528-s001.pdf]

polymers

Self-Organization and Swelling of Ruthenium-Metal Coordination Polymers with PTA (Metal = Ag, Au, Co)

Benjamin Sierra-Martin [1], Manuel Serrano-Ruiz [2], Victoria García-Sakai [3], Franco Scalambra [2], Antonio Romerosa [2] and Antonio Fernandez-Barbero [1,4,*]

1 NanoLab, Department of Chemistry and Physics, University of Almeria, 04120 Almeria, Spain; bsierra@ual.es
2 Inorganic Chemistry Lab-CIESOL, Department of Chemistry and Physics, University of Almeria, 04120 Almeria, Spain; mserrano@ual.es (M.S.-R.); fs649@inlumine.ual.es (F.S.); romerosa@ual.es (A.R.)
3 ISIS Pulsed Neutron and Muon Source, Rutherford Appleton Laboratory, Harwell Science & Innovation Campus, Chilton, Didcot OX11 0QX, UK; victoria.garcia-sakai@stfc.ac.uk
4 Institute of Applied Chemical Sciences, Universidad Autonoma de Chile, 8320000 Santiago, Chile
* Correspondence: afernand@ual.es; Tel.: +34-950-015-909

Table S1. Crystallographic parameters for the coordination polymers Ru-Au, Ru-Ag and Ru-Co.

	Ru-Ag	Ru-Au	Ru-Co
Empirical formula	$C_{19}H_{30}AgCl_2N_6O_2P_2RuS$	$C_{38.5}H_{66}AuN_{17.5}O_4P_4Ru_2$	$C_{39}H_{70}Cl_3CoN_{13}O_2P_4Ru_2S_2$
Formula weight	748.33	1361.07	1308.50
Temperature (K)	273(2)	150(2)	100
Crystal system	orthorhombic	monoclinic	monoclinic
Space group	Pnma	$P2_1/c$	C2/c
a (Å)	11.8733(9)	11.8777(4)	18.4888(13)
b (Å)	12.9333(10)	19.0526(6)	15.9993(12)
c (Å)	17.4306(13)	12.1372(4)	17.5570(13)
α (°)	90.00	90.00	90
β (°)	90.00	110.3910(10)	96.1210(10)
γ (°)	90.00	90.00	90
Volume (Å^3)	2676.7(4)	2574.55(15)	5163.9(7)
Z	4	2	4
ρ_{calc} (g/cm^3)	1.857	1.756	1.683
F(000)	1492.0	1357.0	2676.0
Crystal size (mm)	0.256 × 0.065 × 0.035	0.190 × 0.140 × 0.100	0.182 × 0.063 × 0.058
Radiation	MoKα (λ = 0.71073)	MoKα (λ = 0.71073)	MoKα (λ = 0.71073)
2Θ range for data collection (°)	3.92 to 46.58	4.14 to 50	3.374 to 53.464
Index ranges	$-13 \le h \le 13$, $-14 \le k \le 14$, $-16 \le l \le 19$	$-14 \le h \le 14$, $-20 \le k \le 22$, $-10 \le l \le 14$	$-23 \le h \le 20$, $-20 \le k \le 18$, $-21 \le l \le 22$
Reflections collected	11957	13855	16361
Independent reflections	2035 [R_{int} = 0.0673]	4514 [R_{int} = 0.0203]	5452 [R_{int} = 0.0256]
Data/restraints/ parameters	2035/0/174	4514/0/321	5452/0/301
Goodness-of-fit on F^2	1.114	1.081	1.052
Final R indexes [$I \ge 2\sigma(I)$]	R_1 = 0.0495, wR_2 = 0.0937	R_1 = 0.0246, wR_2 = 0.0602	R_1 = 0.0254, wR_2 = 0.0562
Final R indexes [all data]	R_1 = 0.0696, wR_2 = 0.1035	R_1 = 0.0257, wR_2 = 0.0607	R_1 = 0.0297, wR_2 = 0.0581
Largest diff. peak/hole (e/Å^{-3})	0.70/−0.54	1.12/−0.40	0.44/−0.27

Table S2. Selected bond lengths for Ru-Ag, Ru-Au and Ru-Co.

Ru-Ag		Ru-Au		Ru-Co	
Nuclei	Length (Å)	Nuclei	Length (Å)	Nuclei	Length (Å)
Ru1-P1	2.2856(19)	Ru1-P1	2.2606(8)	Ru2-P1	2.2562(15)
Ru1-S1	2.248(3)	Ru1-N1P	2.027(3)	Ru2-NCN	2.025(5)
Ag1-N1P	2.423(6)	N1P-C1P2	1.152(6)	NCN-CCN	1.140(7)
Ag1-Cl1	2.573(3)	Au1-N3	2.985(3)	Co1-N1	2.260(4)
Ag1-Cl2	2.507(4)	Au1-C1C	2.004(4)	Co1-Cl1	2.3220(14)
		Au1-C2C	1.995(4)	Co1-Cl2	2.3311(14)
		N1C-C1C	1.131(5)	Co1-Cl3	2.3433(14)
		N2C-C2C	1.123(5)		

Table S3. Selected bond angles for Ru-Ag, Ru-Au and Ru-Co.

Ru-Ag		Ru-Au		Ru-Co	
Nuclei	Angle (°)	Nuclei	Angle (°)	Nuclei	Angle (°)
S1-Ru1-P1	92.48(7)	P1-Ru1-P2	98.17(3)	P4-Ru1-P3	94.04(5)
P1-Ru1-P11	93.85(10)	N1P-Ru1-P1	83.22(7)	NCN-Ru1-P3	85.89(14)
C17-S1-Ru1	111.8(2)	N1P-Ru1-P2	86.37(7)	NCN-Ru1-P4	89.08(14)
N1P2-Ag1-N1P	113.0(3)	N1P-C1P-Ru1	175.3(3)	NCN-CCN-Ru1	177.5(5)
Cl2-Ag1-Cl1	124.07(12)	N3-Au1-N3	180.6(2)	N1-Co1-N7′	177.47(16)
N1P-Ag1-Cl1	98.15(14)	N1C-C1C-Au1	179.6(3)	N1-Co1-Cl2	91.66(12)
		N2C-C2C-Au1	179.0(4)	N1-Co1-Cl3	87.97(11)
		C1C-Au1-C1C′	180.00(1)	N1-Co1-Cl1	90.54(12)
		C1C-Au1-C2C	90.02(15)	Cl1-Co1-Cl2	114.36(6)
				Cl1-Co1-Cl3	125.16(6)
